# Progress of Mass Spectrometry-Based Lipidomics in the Dairy Field

**DOI:** 10.3390/foods12112098

**Published:** 2023-05-23

**Authors:** Wei Ren, Mengqi Sun, Xiaoyuan Shi, Tianqi Wang, Yonghui Wang, Changfa Wang, Mengmeng Li

**Affiliations:** School of Agricultural Science and Engineering, Liaocheng Research Institute of Donkey High-Efficiency Breeding and Ecological Feeding, Liaocheng University, Liaocheng 252000, Chinawangcf1967@163.com (C.W.)

**Keywords:** lipidomics, mass spectrometry, dairy products, lipids, application

## Abstract

Lipids play important biological roles, such as providing essential fatty acids and signaling. The wide variety and structural diversity of lipids, and the limited technical means to study them, have seriously hampered the resolution of the mechanisms of action of lipids. With advances in mass spectrometry (MS) and bioinformatic technologies, large amounts of lipids have been detected and analyzed quickly using MS-based lipidomic techniques. Milk lipids, as complex structural metabolites, play a crucial role in human health. In this review, the lipidomic techniques and their applications to dairy products, including compositional analysis, quality identification, authenticity identification, and origin identification, are discussed, with the aim of providing technical support for the development of dairy products.

## 1. Introduction

Humans have consumed cattle and sheep milk for centuries. Dairy products now account for nearly 14% of the global agricultural trade, with cow milk being the most commercially mass-produced product [1,2]. Milk provides more reliable nutrients than other nutritious foods for mammalian infants and children and facilitates human growth and development [3]. Milk fat globules are the main source of dairy lipids, which are an important component of milk [4]. Lipids store energy for the body, participate in signal transduction, material transport, cell development, differentiation, and apoptosis, regulate lipid metabolism and the intestinal flora composition, alleviate inflammatory responses, and promote neurodevelopment [1,2]. In addition, the milk fat globular membrane has potential effects on cardiovascular disease, bone loss, inflammation, skin conditions, infections, age-related cognitive decline, and muscle loss [4,5,6]. Research found that joint space and bone erosion were significantly restored after treatment of collagen-induced arthritis (CIA) mice with camel milk lipids by inhibition of the nuclear factor kappa B (NF-κB) pathway [7]. The lipid concentrations in sheep milk, goat milk, and cow milk are 40–99 g/L, 41–45 g/L, and 33–47 g/L, respectively [8,9]. Milk lipids are mainly composed of 98–99% triglyceride (TG), 0.3–0.8% phospholipid (PL), and 0.3–0.5% cholesterol (Chol), of which about 65% of TG and PL are located in the milk fat globule membrane [10]. Over 200 fatty acids (FAs) and 400 TGs were found in human milk, and over 400 FAs were found in cow and goat milk [11,12]. With their complex structure, variety, and a large number, milk lipids are considered to be the most complex substances in nature in terms of their composition. Analytical methods fail to comprehensively characterize lipid molecules, making it challenging to examine their metabolic pathways and functional energy regulation in depth. With the recent introduction of the concept of “Omics” and the important biological functions of lipids, lipidomics has become a cutting-edge research technique, making important contributions to the resolution of milk quality and milk lipid function [13,14]. Mass spectrometry (MS) is a method to define the relative molecular weight and structure of samples by determining the mass number of molecular ions and fragments in the sample. MS has the advantages of high throughput, sensitivity, and accuracy. MS-based lipidomics has focused on the lipid analysis of milk, which has largely resolved the complexity of lipids [15]. High-performance liquid chromatography-atmospheric pressure chemical ionization-mass spectrometry/mass spectrometry (HPLC-APCI-MS/MS) was found to provide a comprehensive characterization of TG molecules and individual regional isomers in milk lipids [16]. Matrix-assisted laser desorption ionization-time of flight MS (MALDI-TOF MS) can be used to identify non-cow milk from cow milk and to characterize milk lipids in cheese and other dairy products [17,18]. Gas chromatography-MS (GC-MS) has been widely used in the separation and identification of milk FAs [19].

This review provides an overview of MS-based lipidomics techniques and their applications to milk and milk products, including compositional analysis, quality identification, authenticity identification, and origin identification. We aim to provide a scientific reference for further application of lipidomic research into the composition and function of lipids in dairy products, technical support, and a theoretical basis for the healthy development of the dairy industry.

## 2. Classification and Function of Lipids

**Figure 1:**
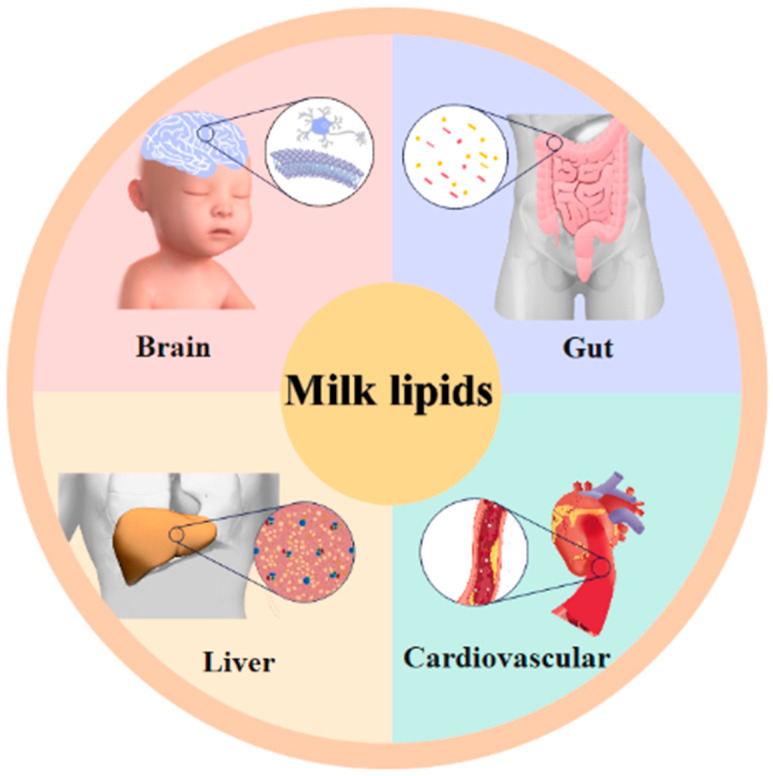
The main functions of milk lipids.

The biology of lipids, as vital biomolecules in the structure of living organisms, is generally considered to involve a group of enzymes, binding proteins, and receptors that act in concert to produce lipids, which are soluble in organic solvents [20]. Lipids are divided into eight classes based on their chemical structure: FAs (e.g., linoleic acid and linolenic acid), glycerolipids (GLs, e.g., diacylglycerol and TG), glycerophospholipids (GPs, e.g., phosphatidylcholine and phosphatidylethanolamine) sphingolipids (SPs, e.g., ceramide and sphingomyelin), sterol lipids (STs, e.g., cholesterol), saccharolipid (SLs, e.g., digalactosyldiacylglycerol), prenol lipids (PRs, e.g., carotenoid) and polyketides (PKs) [21,22,23]. As shown in Figure 1, 98–99% of TGs and 0.3–0.8% of PLs in milk lipids are involved in the regulation of lipid metabolism and the intestinal flora, the reduction of inflammatory factors, and the prevention of cardiovascular disease. Sphingolipids, which are strongly linked to neurodevelopment, are the second most significant membrane lipids after phospholipids.

TGs comprise a glycerol backbone and three FAs. The main palmitic acids in the sn-2 position of TGs are 1,3-UFA-2-palmitic acid triglyceride (UPU, over 70%), 1(3)-UFA-2-palmitic acid-3(1)-SFA (UPS, 20%), and 1,3-SFA-2-palmitic acid (SPS, 5%) [11,24]. Free palmitic acid is not directly absorbed by the epithelial cells of the small intestine; therefore, it combines with divalent cations (calcium and magnesium) to form insoluble salts under alkaline conditions, thus reducing stool hardness in infants and reducing bone mineralization [25,26,27]. Mice fed with human milk, donkey milk, and cow milk (palmitate in the sn-2 position in TGs) were found to have uncoupled mitochondria, thus controlling skeletal muscle mitochondrial dynamics by raising the redox state of skeletal muscle, and reducing inflammatory factors and skeletal muscle lipid accumulation, which improves insulin tolerance, and regulates lipid metabolism by increasing the level of N-oleoylethanolamine in the liver and muscle [28]. *Apoe* (encoding apolipoprotein E) knockout mice were fed a high-fat diet of medium-and long-chain triacylglycerols, which reduced the intestinal flora ratio of Firmicutes to Bacteroidetes, increased the abundance of short-chain fatty acid bacteria, and decreased the abundance of *Ruminococcus torques*, *Ruminiclostridium 9*, *Catenibacterium*, and *Eubacterium fissicatena*, thus alleviating atherosclerosis induced by a high-fat diet [29]. In short, TGs in milk facilitate the digestion and absorption of lipids and calcium in newborns and young children; decrease inflammation, stool hardness, fat accumulation, and improper lipid metabolism caused by high-fat meals; and regulate the composition of the gut flora.

Sphingomyelin (SM), as an important structural component of neurons and lipid bilayers, is the main SP in milk (25–35% of the polar lipids) [30,31]. In a previous study, SM decreased the absorption of Chol, TG, and long-chain fatty acids (LCFAs) in the intestines of rodents by inhibiting intestinal bacterial-derived lipopolysaccharide (LPS) translocation and/or inhibition of its pro-inflammatory effects to affect the intestinal-liver axis, which regulates blood lipids and prevents non-alcoholic fatty liver disease associated with obesity [32]. Milk SM (MSM) attenuated hepatic steatosis (via reduced TGs), hepatomegaly, hyperlipidemia, and adipose tissue inflammation in obese mice fed a high-fat diet [33]. Meanwhile, C57BL/6J mice fed MSM showed an increase in the expression of glucose transporter type 4 mRNA, hepatic sterol regulatory element binding transcription factor 2 mRNA, and HMG-CoA reductase mRNA, and reduced the inflammatory response caused by interleukin-6 mRNA expression, which caused a 47% reduction in TG levels in the liver, modified the distal intestinal flora (gram-negative bacteria significant decreased, bifidobacteria significant increased), and reduced serum PS levels [34]. MSM inhibited the absorption of fat and cholesterol in the intestine of rats more strongly than eggs, which was caused by the stronger hydrophobic interactions between MSM and other lipids [35]. Cer and Sph, the hydrolysis products of MSM, reduced LPS levels in the blood and systemic inflammation via inhibition of pro-inflammatory gene expression in RAW 264.7 macrophages [36]. Moreover, Sph has in vitro bactericidal properties and alleviates intestinal malnutrition and inflammatory reactions [37]. In short, MSM and its hydrolysis products work to improve the abnormal lipid metabolism, intestinal barrier dysfunction, inflammatory response, and weight gain caused by high-fat diets.

## 3. MS-Based Lipidomic Technologies

**Figure 2:**
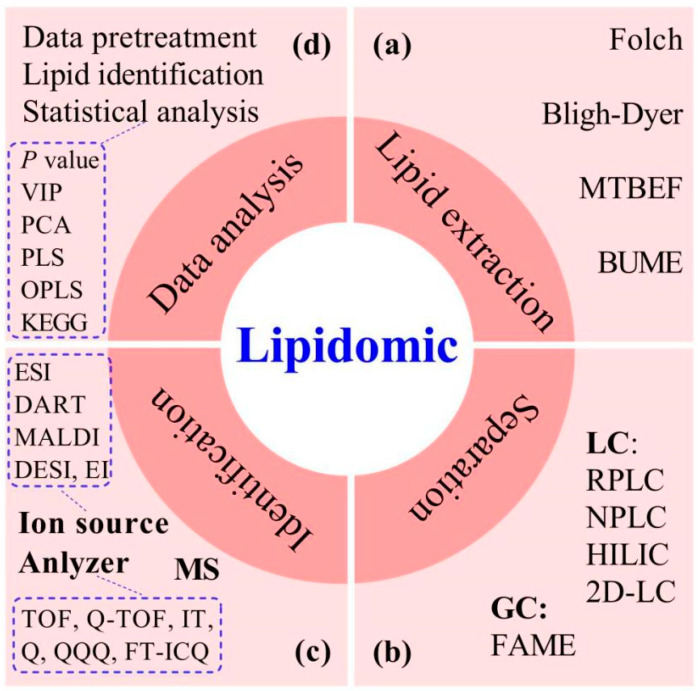
The workflow of MS-based lipidomics (**a**–**d**). APCI, atmospheric pressure chemical ionization; APPI, atmospheric pressure photoionization; BUME, butanol/methanol; DART, direct analysis in real time; DESI, desorption electrospray ionization; DI, direct injection; 2DLC, two-dimensional liquid chromatography; EI: electron impact ionization; ESI, electrospray ionization; FT-ICQ, Fourier transform ion cyclotron resonance; GC, gas chromatography; HILIC, hydrophilic interaction liquid chromatography; LAESI, laser ablation electro spray ionization; LC, liquid chromatography; MALDI, matrix-assisted laser desorption ionization; MTBE, methyl tert-butyl ether; NPLC, normal phase liquid chromatography; RPLC, reversed phase liquid chromatography; TOF, time-of-flight; Q, quadrupole; QQQ, triple quadrupole.

As a subset of metabolomics, lipidomics comprises the study of the molecular properties of lipids in organisms, tissues, or cells. Lipidomics is advantageous for comprehensive and systematic research into lipid structure, metabolic pathways, and functions, including lipid-related protein expression and gene regulation [15,38]. As shown in Figure 2, lipidomics mainly includes lipid extraction, isolation, identification, and data analysis.

### 3.1. Lipid Extraction

The process of lipid extraction from biological samples is inseparable from lipid analysis and commonly includes Folch extraction [39], Bligh extraction [40], methyl-tert-butyl ether (MTBE) extraction [41], butanol-methanol (BUME) extraction [42], solid phase extraction (SPE) [43], and supercritical fluid extraction (SFE) [44]. Folch extraction was the earliest proposed chloroform-methanol system, which can efficiently extract both free and bound lipids but is highly toxic and unsuitable for food lipid extraction [39]. Bligh extraction uses less solvent, extracts faster, and recovers more lipids [45]. Lipids are dissolved in the MTBE organic phase to protect unstable lipids from degradation and avoid the use of chloroform [41]. For BUME, the butanol content is not readily evaporated [45]. Convenient operation and reduced consumption of reagents are hallmarks of SPE, which is used to extract liquid-phase lipids and reduce the specific enrichment of lipids [46]. SFE is based on the difference in solubility of different compounds in SF and has recyclable extractants [44].

### 3.2. Isolation and Identification

Currently, targeting is the accurate analysis of one or several classes of target lipids, whereas the analysis of lipid profiles in biological samples uses non-targeted methods [47]. Separation methods include GC, liquid chromatography (LC), supercritical fluid chromatography (SFC), hydrophilic interaction chromatography (HILIC), and capillary electrophoresis (CE) [48,49,50]. GC is often used to analyze the composition of milk FAs; however, it requires derivatization or primary separation by thin-layer chromatography (TLC), followed by further analysis by GC, which is time-consuming [51]. However, when using GC analysis alone, 200 m SLB-IL111 columns are the first choice for a more complete analysis of all FAs in cow and sheep milk [12]. Furthermore, LC is applied to biomolecules that have a high relative molecular weight, are difficult to vaporize, are non-volatile, and are heat-sensitive. Examples of LC include reversed-phase high-performance liquid chromatography (RP-HPLC), normal-phase high-performance liquid chromatography (NP-HPLC), silver-ion liquid chromatography, and chiral high-performance liquid chromatography. TLC can separate lipids intuitively and quickly but has limited throughput, and weak sensitivity and resolution, which can disrupt lipid structure [48,49]. SFC provides a high-resolution, rapid, and comprehensive analysis of mixed lipids and improves the elution capacity of the mobile phase by varying its density [52]. HILIC is applied to the separation of polar lipids [53]. Nuclear magnetic resonance sensitivity is inadequate to detect low-abundance lipids [54]. Furthermore, MS, which consists of an injection system, an ion source, a mass analyzer, a detector, and an amplifier recorder, is also the most common approach for lipid identification. The injection system is separated into direct injection (e.g., diffusion injection) and indirect injection (e.g., probe injection and chromatographic injection) [55]. Electron bombardment ionization sources (EI), electrospray ionization (ESI), APCI, atmospheric pressure photoionization (APPI), and MALDI are the most common ion sources [46]. The main mass analyzers are quadrupole (Q), time of flight (TOF), ion trap, and electrostatic field orbital trap (Orbitrap) [56,57,58].

Tandem MS technology has received considerable emphasis and development to realize the in-depth analysis of lipids. GC-MS can be used to determine whether cow milk contains vegetable milk (β-sitosterol) [59]. The quantitative study of lipids, FAs, and region-specific sites is possible using the LC-MS approach, whereas the HPLC-ESI-MS quantification approach requires no derivatization and offers precise relative molecular mass and structure information, although it takes longer to analyze [60]. HPLC-APCI-MS/MS is used for the complete identification of TG molecules in milk lipids and the identification of individual regional isomers [16]. HPLC-evaporative light-scattering detection (ELSD) is suitable for the analysis of the main lipid classes in dairy products [8]. Research found that HPLC-ELSD was suitable for the quantitative analysis of PLs in human milk and infant formula. Qualitative analysis of PL categories in human milk can be achieved using HILIC-TOF-MS, while further quantitative analysis of the identified PL structures can be undertaken using HILIC-MS/MS [61]. MALDI-TOF-MS is commonly used for the rapid screening of polar and non-polar lipids, and can be used to identify milk and non-milk samples by comparing the mass spectra generated with those in the corresponding reference databases; however, it lacks the quantitative analysis of the full range of lipids [17,62].

### 3.3. Data Analysis

Data analyses include pre-processing (filtering, alignment, peak identification, lipid annotation, and normalization), lipid classification, multivariate statistical analysis, such as the P value, variable importance in projection (VIP), principal component analysis (PCA), partial least squares regression (PLS), and orthogonal partial least squares (OPLS), differential lipid screening, differential lipid statistical analysis, cluster analysis, and functional enrichment. Lipid molecule data are routinely analyzed using software such as Lipid View, LipidXplorer, MZmine, and XCMS, as well as databases such as CyberLipids, the Human Metabolome Database, the Kyoto Encyclopedia of Genes and Genomes, LIPIDAT, the Human Metabolome Database, Lipid Bank, Lipid Maps, Lipid Library, and LipidBlast [15].

## 4. Application of MS-Based Lipidomics in the Dairy Industry

**Figure 3:**
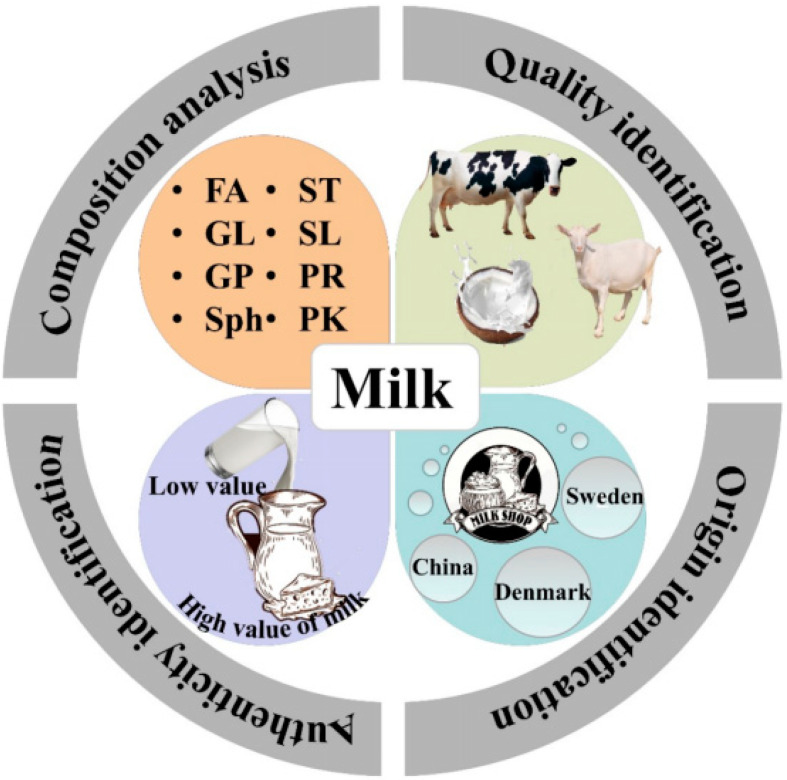
MS-based lipidomics in dairy products. FA, fat acid; GL, glycerolipid; GP: glycerophospholipid; Sph: sphingolipid; ST, sterol lipid; SL, saccharolipid; PR, prenol lipid; PK, polyketide.

As shown in Figure 3, research on the application of lipidomic techniques in the analysis of milk and product composition, quality identification, authenticity identification, and origin identification has provided technical support for improving milk quality and the healthy development of the dairy industry.

### 4.1. Composition Analysis

As shown in Table 1, statistical lipid profiles based on lipidomics in dairy products facilitate the further development of lipidomics in milk. Milk is one of the most complex natural lipid mixtures [11]. In human milk, among a total of 478 lipids, 223 TGs, 26 diglycerides (DGs), 59 PCs, 49 PEs, 18 phosphatidylglycerols (PGs), 16 PIs, eight phosphatidic acids (PAs), three phosphatidylglycerols (PSs), 11 cardiolipins (CLs), one Sph, nine ceramides (Cers), 30 SMs, five hexosylceramides (HexCers), and 16 bishexosylceramides (Hex2Cers) were identified using ultra-high-performance liquid chromatography (UHPLC)-Q-TOF-MS [63]. In cow milk, 335 lipid molecules species, including 114 TGs, 15 DGs, nine CLs, seven PAs, 70 PCs, seven PEs, 20 PGs, six PSs, eight Cers, 22 SMs, six HexCers, and 18 Hex2Cers, were identified using UHPLC-Q-TOF-MS [64]. In another study, 3454 TG molecules belonging to 220 TG groups (36 saturated TGs, 37 monounsaturated TGs, 37 di-unsaturated TGs, and 110 polyunsaturated TGs) were identified in cow milk using LC-MS [65]. In Ultra-High temperature (UHT) and reconstituted milk (whole milk), 577 lipid molecules, including 312 TGs, 16 DGs, two monoglycerides (MGs), 43 PCs, 65 PEs, eight PGs, nine PIs, two PSs, nine lysophosphatidylcholines (LPCs), six lysophosphatylethanolamines (LPEs), 12 CLs, 14 Cers, six glycosylceramides, 12 diacylglycerophosphoethanolamine glycans, 26 SMs, three Wax esters (WEs), one stearamide, and Sphingosine (So) were identified using ultra-performance liquid chromatography (UPLC)-Q-Exactive Orbitrap-MS [66]. In goat milk, a total of 756 lipid molecules in 14 lipid subclasses (five CLs, 45 Cers, 17 LPCs, four LPEs, 36 PCs, 80 PEs, nine PGs, 22 PIs, two acyl carnitines (AcCas), 55 HexCers, 56 SMs, 17 FAs, 15 DGs, and 416 TGs) were identified using UPLC-Q-Exactive Orbitrap-MS [67]. Ewe milk was identified to comprise 98.11% TGs, 1.45% Chol + DG + free FA, 0.02% CEs, 0.03% MGs, 4.98% Lactosylceramides (LacCers), 32.65% PEs, 4.16% PIs, 4.96% PSs, 27.21% PCs, and 26.05% SMs using HPLC-ELSD [8]. In addition, a total of 335 lipid molecules belonging to 13 subclasses (4.48% DGs, 34.03% TGs, 2.69% CLs, 2.09% PAs, 9.85% PCs, 20.90% PEs, 2.09% PGs, 5.97% PIs, 1.79% PSs, 2.39% Cers, 1.79% HexCers, 5.37% Hex2Cer, and 6.57% SMs) were identified in donkey milk using UPLC-MS/MS [68]. Chol, Cer, PL (PI, PE, PS, PC, SM, GluCer, and LacCer), with the highest Cer content, especially LacCer, were identified in the polar lipids of Amiata donkey milk using HPLC-ESI-MS [69]. The GC-flame ionization detector (GC-FID) analysis of the milk lipids of the American quarter horse revealed saturated fatty acids (SFAs) (C4:0, C6:0, C8:0, C10:0, C12:0, C14:0, C16:0, C18:0) and unsaturated fatty acids (UFAs) (C14:1, C16:1, C18:1n9cis, C18:2n6cis, C20:1n9, C18:3n3, C20:2n6, C20:3n3) [70]. Notably, dairy products from camels and horses have not been documented for their lipid profiles using lipidomics techniques.

Milk-like products extracted from plants (hereafter referred to as “plant-based milk alternatives”) are higher in dietary fiber and lower in fat than animal milk and contain a diverse variety of lipids to compensate for this lack of fat (e.g., oleic acid, linoleic acid, and medium- and long-chain FAs). Analysis of soybean milk using UPLC-Q-Exactive Orbitrap-MS revealed one PC, one PE, one DG, seven PAs, nine PGs, and four PIs, and a ratio of 1:0.8:0.4 for SFA, monounsaturated fatty acid (MUFA), and polyunsaturated fatty acid (PUFA) [77]. Interestingly, the majority of plant-based milk alternatives (e.g., walnut, almond, coconut, peanut, and oat milk) have not been documented for their lipid contents using lipidomic analysis, implying that more research is needed.

### 4.2. Milk Quality Identification

As shown in Table 2, the lipid composition of milk varies between species, ages, lactation periods, or seasons, and the lipid composition of different kinds of milk and milk products is compared and analyzed to obtain their corresponding biomarkers. Research found that in the analysis of human milk lipids during the growth trajectory of infants, medium-chain sphingolipids [SM (d18:0/12:0)], phospholipids containing dihomo-γ-linolenic acid and docosahexaenoic acid (DHA) [PE (20:3/22:6)], hydroxylated lipids derived from dihomo-γ-linolenic acid, oleic acid-containing TG [TG (16:0/17:1/18:1), and TG (18:0/18:1/18:1)], and medium-chain SFAs were defined as biomarkers using LC-High-Resolution-MS [78]. Researchers discovered that bovine milk has significantly higher levels of PE and significantly lower levels of PC and SM; goat milk has significantly higher levels of polar lipids (LacCer and PI); and sheep milk has significantly lower levels of total Chol, DG, and free FA (FFA), as assessed using HPLC-ELSD [8]. In human, cow, and goat milk, TG, DG, SM, PC, Cer, HexCer, Hex2Cer, PE, PG, PS, PI, PA, and CL belong to 13 lipid subclasses, among which 326 lipid molecules were defined as potential biomarkers; 16 TGs and 10 DGs were present in significantly higher levels in bovine milk, and 12 TGs were present in significantly higher levels in goat milk, and only DG (20:2/20:2) levels were significantly higher in human milk samples, as assessed using UPLC-QTOF-MS [79]. In another study, 39 significantly different lipid molecules (13 TGs, 12 DGs, three HexCers, and polarity lipids) were identified between human milk and cow milk; 14 significantly different lipid molecules (one PC, one DG, and 12 TGs) between human milk and goat milk, and only three different TGs between cow milk and goat milk using UPLC-QTOF-MS [74]. A total of 63 different lipid molecules—among which PE (16:0/22:5) and PE (16:0/20:4) were upregulated, and Hex2Cer (d14:0/24:0) and Hex2Cer (d15:0/24:1) were downregulated—were identified in cow colostrum milk and milk using UHPLC-Q-TOF-MS [80]. Differences in TGs of pasteurized, homogenized, and freeze-dried cow and buffalo milk were revealed using UPLC-MS, which showed that pasteurization, homogenization, and freeze-drying did not significantly affect the TG composition of cow and buffalo milk [81]. In a comparison of donkey colostrum milk with donkey milk using UPLC-MS/MS, 60 lipid molecules (22 PEs, 22 PEs, 12 TGs, eight SMs, four PCs, three PAs, three Hex2Cers, two CLs, two PIs, two Cers, one HexCer, and one PG) had significantly different abundances, among which 17 were upregulated and 43 were downregulated, and thus were considered as potential lipid markers [68]. A total of 15 biomarkers were identified in UHT and recovered milk (whole milk) using UPLC-Q-Exactive Orbitrap-MS, with higher levels of PE (16:0/18:1), PE (18:0/18:1), PE (18:1/18:1), PE (18:1/20:3), PE (18:1/14: 0), TG (6:0/14:1/18:3), TG (15:0/14:0/10:0), PC (18:0/18:1), LPE (18:0), CL (18:1/18:0/18:0/18:1), 16-hydroxyhexadecanoic acid, and 12-hydroxydecanoic acid in the UHT milk; and higher levels of TG (16:0/8:0/18:1), TG (6:0/18:0/18:1), and PE (16:0/18:2) in the recovered milk [66]. An analysis of colostrum and standing milk lipids from the American Quarter Horse using GC-FID revealed higher levels of SFA (palmitic, capric, lauric, and myristic acids) in colostrum and higher levels of UFA in standing milk [70]. Theoretically, comparative identification of potential lipid markers provides an effective method to identify milk types; however, in practice, we lack lipid markers for donkey milk, camel milk, and plant-based milk alternatives.

### 4.3. Milk Authenticity Identification

The dairy industry has undergone rapid growth, and market competition has become increasingly fierce, leading businesses to blend low-cost milk with high-cost milk or mix plant-based milk alternatives with animal milk to increase profits. This not only harms the quality of dairy products but also the health of consumers. Common adulteration detection techniques include specific gravity determination, freezing point determination, dry matter determination, diphenylamine determination, electrophoresis, casein precipitation, color development, MS, and chromatographic methods [86]. However, immunological techniques, PCR, and plate gel electrophoresis were incapable of detecting the adulteration of cow milk in sheep cheese [87]. Subsequently, the advent of GC-MS technology mostly solved the challenges of detecting the adulteration of cow milk with vegetable lipids (β-sitosterol) or other kinds of animal milk [59]. Analysis of goat, soy, and cow milk using UPLC-Q-Exactive Orbitrap-MS showed that soy milk was enriched in linoleic acid (LA), a-linolenic acid (ALA), PE, PG, PS, and PC; cow milk was enriched in Cer and DG; and goat milk was enriched in medium chain TG, UFA, n-6 FA, and n-3 FA, especially eicosapentaenoic acid (EPA) and DHA, among which DHA, EPA, SFA, PUFA, MUFA, medium chain triglycerides, arachidonic acid (ARA), ALA, LA, TG, DG, Cer, PG, PC, and LPC were potential lipid biomarkers to differentiate goat milk, soy milk, and cow milk [77]. TGs were most abundant in coconut milk [TG (32:0), TG (34:0), TG (36:0), TG (38:0), and TG (40:0)], whereas PC (28:0), PC (30:0), PC (32:0), PC (34:1), and PC (36:1) dominated cow milk, and soy milk had the highest levels of PC (34:1), PC (36:3), PC (37:3), and PC (39:5), which were identified using MALDI-TOF-MS [18]. The lipid profile markers of bovine, sheep, goat, buffalo, and mixed (80% bovine, 10% sheep, and 10% buffalo) cheeses were revealed as TG (18:6), TG (58:7), and TG (58:0) for bovine and sheep cheeses; PC (40:3), TG (52:3), and TG (54:5) for buffalo cheese, diacylglycerophosphate (32:1), PE (30:1), and PE (38:1) for goat cheese, and glycerophosphoglycerol (33:2) glycerophosphoglycerol (36:4), and PC (34:0) for mixed cheese using MALDI-Direct Imprinting in Glass Surface (DIGS)-MS [88]. Dodecylbenzene sulfonate is a lipid marker for the adulteration of infant formula with detergent powder, as assessed using LC-QTOF-MS [89].

### 4.4. Identification of the Origin of Dairy Products

Consumers are becoming more inclined to track the origin of dairy products because of differences in milk composition between regions, in addition to concerns surrounding food safety and counterfeiting. Interestingly, lipidomics plays a vital role in determining the origin of milk and has promising application potential. Hence, the six TGs recognized by all countries (TG 18:1/18:1/18:1, 18:1/16:0/18:2, 18:1/16:0/18:1, 18:1/18:0/18:1, 16:0/18:1/16:0, and 18:0/16:0/18:1); the unique TGs 16:1/18:1/18:2, 16:1/18:1/18:1, and 16:1/18:2/18:0 containing hexadecenoic acid identified in Spanish and Finnish human milk; TGs 10:0/10:0/10:0, 14:0/14:0/14:0 in American human milk; and TGs 10:0/18:1/10:0, 10:0/16:0/18:2, 12:0/10:0/12:0, 12:0/18:1/12:0, 18:2/12:0/10:0, 18:2/12:0/12:0, 18:2/14:0/18:2, 18:2/14:0/12:0, 18:2/16:0/10:0, 18:2/16:1/18:2, 18:2/16:0/16:1, 18:1/14:0/16:1, and 18:1/16:0/10:0 containing linoleic acid (C18:2) in Chinese human milk have been identified [90]. 18:1/16:0/18:1, 18:1/16:0/18:2, and 18:1/18:1 had the highest proportions in Guangzhou, Beijing, and Chengdu, respectively, and LC-MS/MS identified the best balance of TGs in Jinhua and Zhengzhou [90]. UPLC-ESI-MS analysis revealed that TGs 18:1/16:0/18:1 (9.4% of total TGs), 18:2/16:0/18:1 (5.4%), 18:1/16:0/12:0 (3.5%) 18:1/16:0/18:0 (3.2%), 18:1/14:0/18:1 (3.2%), 18:1/16:0/16:0 (3.0%), 18:1/12:0/18:1 (2.5%), and 18:1/18:1/18:1 (2.5%) were abundant in Finnish human milk; and TGs 8:2/16:0/18:1 (10.3%) 18:1/16:0/18:1 (7.1%), 18:2/16:0/18:2 (4.5%), 18:2/18:2/18:1 (3.3%), 18:1/16:0/18:0 (2.4%), 18:2/18:1/18:1 (2.4%), 18:1/16:0/16:0 (2.4%), 18:2/12:0/18:1 (2.2%), and 18:1/16:0/12:0 (2.1%) were abundant in Chinese human milk. Notably, the ratios of oleic acid (18:1n-9) and myristic acid (14:0) to total FAs were higher in Finnish human milk than in Chinese human milk, yet the ratios of C18 FA and linoleic acid (18:2n-6) to total FAs were lower [91]. In Chinese human milk, higher levels of FAs (C18:1, C24:1, ARA, and DHA) were identified by GC-MS, yet the ratios of n-6/n-3 and LA/ALA were higher in Egyptian human milk [92]. A total of 51 significantly different lipids belonging to five subclasses were identified in goat milk from three farms in Yunnan, Shaanxi, and Shandong, of which 38 lipids were considered potential lipid markers for origin identification, with Yunnan goat milk having higher Cer, PC, and SM levels and lower PI and FA contents [67].

## 5. Conclusions

In the dairy field, MS-based -lipidomics research has been widely used to analyze milk lipids in recent years, which provides the theoretical and data basis for the development of milk lipid function. This review summarized milk lipid compositions and lipid biomarkers determined using MS-based lipidomics, which showed that lipids were different in kinds of milk from different species or different regions. This will contribute to the identification of milk quality, safety issues, and traceability of milk origin. However, the complex lipid profile of milk and the limited exploitation of special kinds of animal milk, especially camel, donkey, horse, and plant-based milk alternatives deserve more research attention. Therefore, the application of MS-based lipidomics to a more comprehensive range of milk types should be prioritized.

## Figures and Tables

**Table 1:** Lipid composition of animal and plant-based milk alternatives.

Item	Technology	Lipid	References
Animal Milk			
Human milk (Jilin Province, China)	UHPLC-Q-TOF-MS	478 lipids (26 DGs, 223 TGs, 59 PCs, 49 PEs, 18 PGs, 16 PIs, 8 PAs, 3 PSs, 11 CLs, 5 Sphs, 9 Cers, 30 SMs, 5 HexCers, 16 Hex2Cers).	[63]
Human Colostrum (Poland)	LC–Q-TOF–MS	701 lipids, 443 FAs, 134 TGs, 6 TGOs, 13 DGs, 3 MGs, 32 SMs, 1 PAs, 13 PEs, 5 PEOs, 38 PCs, 2 PCOs, 8 PIs, 3 PSs.	[71]
Human milk	LC–Q-TOF–MS	617 lipids, 304 TGs, 204 DGs, 14 MGs, 34 GPs, 13 Cers, 3 Sphs, 5 Neutral glycolsphingolipids, 6 Phosphosphingolipids, 5 Prenol lipids, 29 Sterol lipids.	[72]
	RP-LC-MS/MS	237 lipids, 118TGs, 10 DGs, 22 PCs, 5 PSs, 13 PEs, 8 PIs, 6 LPCs, 6 LPEs, 30 SMs, 6 HexCers, 8 Cers, 5 GMs	[73]
Bovine milk	UHPLC-Q-TOF-MS	335 lipids, 9 CLs, 7 PAs, 70 PCs, 7 PEs, 20 PGs, 6 PSs, 8 Cers, 22 SMs, 6 HexCers, 18 Hex2Cers, 15 DGs, 114 TGs.	[64]
	HPLC-ELSD	97.75% TG, 1.81% Chol+DG+FFA, 0.04% CE, 0.04% MG, 5.10% LacCer, 36.58% PE, 6.18% PI, 7.28% PS, 24.60% PC, 20.25% SM.	[8]
Ultra-high temperature and reconstituted milk (Bovine milk)	UPLC-Q-Exactive Orbitrap-MS	577 lipids, 312 TGs, 16 DGs, 2 MGs, 43 PCs, 65 PEs, 8 PGs, 9 PIs, 2 PSs, 9 LPCs, 6 LPEs, 12 CLs, 14 Cers, 6 CerG1s, 12 CerG2s, 26 SMs, 3 WEs, 1 stearamides, 1 Sos.	[66]
Powder buttermilk (Bovine milk)	HPLC-ELSD	54.55% TG, 10.51% Chol+DG+FFA, 1.73% MG, 1.50% CE, 0.30% PA, 19.80% PE, 4.93% PI, 20.60% PS, 33.91% PC, 16.87% SM, 1.22% GluCcer, 2.37% LacCer.	[8]
Goat milk	UPLC-QTOF-MS	103 lipids, 17 PCs, 15 SMs, 11 Cers, 3 HexCers, 25 TGs, 32 DGs.	[74]
	UPLC-Q-Exactive Orbitrap-MS	756 lipids in 14 lipid subclasses, 5 CLs, 45 Cers, 17 LPCs, 4 LPEs, 36 PCs, 80 PEs, 9 PGs, 22 PIs, 2 AcCas, 55 HexCers, 56 SMs, 17 FAs, 15 DGs, 416 TGs.	[75]
	HPLC-ELSD	97.32% TG, 1.89% Chol+DG+FFAs, 0.04% CE, 0.10% MG, 7.57% LacCer, 29.17% PE, 5.77% PI, 7.65% PS, 26.25% PC, 23.24% SM.	[8]
Ewe milk	HPLC-ELSD	98.11% TG, 1.45% Chol+DG+FFAs, 0.02% CE, 0.03% MG, 4.98% LacCer, 32.65% PE, 4.16% PI, 4.96% PS, 27.21% PC, 26.05% SM.	[8]
Donkey milk	UPLC-MS/MS	335 lipids in 13 lipid subclasses, 4.48% DG, 34.03% TG, 2.69% CL, 2.09% PA, 9.85% PC, 20.90% PE, 2.09% PG, 5.97% PI, 1.79% PS, 2.39% Cer, 1.79% HexCer, 5.37% Hex2Cer, 6.57% SM.	[68]
	HILIC-ESI-MS/MS	157 PLs (11 LPEs, 8 LPCs, 40 PIs, 20 PSs, 25 PEs, 41 PCs, 12 SMs) and 25 HexCers (11 HexCers, 10 Hex2Cers, 4 Hex3Cers).	[76]
Horse milk	GC-FID	SFA (C4:0, C6:0, C8:0, C10:0, C12:0, C14:0, C16:0, C18:0), USFA (C14:1, C16:1, C18:1n9cis, C18:2n6cis, C20:1n9, C18:3n3, C20:2n6, C20:3n3).	[70]
**Plant-based milk alternatives**			
Soya milk	UPLC-Q-Exactive Orbitrap-MS	1 PC, 7 PAs, 1 PE, 9 PGs, 4 PIs, 1 PS, 1 DG, ALA and LA, SFA: MUFA: UFA = 1:0.8:0.4.	[77]

Abbreviations: FA, fatty acid; SFA, saturated fatty acid; UFA, unsaturated fatty acid; MUFA, monounsaturated fatty acid; PUFA, polyunsaturated fatty acid; TG, triglyceride; PL, phospholipid; GL, glycerolipid; GP, glycerophospholipid; Sph, sphingolipids; LPC, lysophosphatidylcholine; LPE, lysophosphatidyl ethanolamine; PC, phosphatidylcholine; PE, phosphatidyl ethanolamine; PA, phosphatidic acid; PA-P, PA-plasmalogen; PI, phosphatidylinositol; PS, phosphatidylserine; PG, phosphatidylglycerol; Cer, ceramide; SM, sphingomyelin; MG, monoglyceride; DG, diglyceride; TG, triglyceride; HexCer, hexosylceramide; Hex2Cer, bishexosylceramide; Hex3Cers, trihexylglyceramide; WE, wax ester; So, sphingosine; LA, linoleic acid; ARA, arachidonic acid; ALA, a-linolenic acid; EPA, eicosapentaenoic acid; DHA, docosahexaenoic acid; GluCcer: glucosylceramide; LacCer: lactosyl-ceramide; CE: cholesteryl ester; CL, cardiolipin; CerP, ceramide phosphate; GlcCer, glucosylceramide; Chol, cholesterol; LC-PUFAs, long-chain polyunsaturated fatty acids; TGO, ether analog of triacyglycerol; RP-LC, reversed-phase liquid chromatography; MS, mass spectrometry; LC, liquid chromatography; HPLC, high-performance liquid chromatography; UPLC, ultra-performance liquid chromatography; UHPLC, ultra-high performance liquid chromatography; GC, gas chromatography; HILIC, hydrophilic interaction chromatography; ESI, electrospray ionization; FID, flame ionization detector; ELSD, evaporative light-scattering detection; Q, quadrupole; TOF, time of flight; Orbitrap, electrostatic field orbital trap.

**Table 2:** Comparison of lipids in different milks.

Item	Technology	Characteristics of Lipid	References
Human milk (premature birth and normal)	LC-MS/MS	PC and PE increased significantly, SM, TG (14:0/18:2/18:3), PE (17:2/22:6) and glycerol phosphate ethylenediamine analog significantly decreased.	[82]
Human milk (babies grow faster and normal)	LC-High-Resolution-MS	MCSAT, SM and phosphatidylethanolamine are significantly increased, oleic acid (C18:1) TG and DGLA-derived oxidized lipids are significantly decreased.	[78]
Human milk and Infant formula milk	UHPLC-Q-TOF-MS	97 different lipids, 10 TGs, 4 DGs, 27 PEs, 19 PCs, 3 PGs, 11 PIs, 1 PA, 1 Sph, 1 CL, 7 SMs and 13 HexCers.	[63]
	LC–Q-TOF–MS	Four potential biomarkers (MCSAT, oleic acid, EPA, and DHA), 46 postnatal growth markers in IFM. The unique lipids species TG 66:18 and PI 34:2 in IFM, and PE-O 36:5, PE-O 38:5 in HM.	[71]
	UPLC-Q-TRAP-MS	28 different lipids (2 FAs, 2 DGs, 8 TGs, 1 LPC, 3PEs, and 4 SMs).	[7]
Human milk, Infant formula milk and Bovine milk	SPME-LC-MS	72 unique lipids in HM (58 TGs, 9 DGs, 1 PC, 4 SMs), 48 unique lipids in IFM, and 58 unique lipids in BM.	[72]
Human milk and Donkey milk	RP-HPLC-APCI-MS	72 different TGs, POO (14.1%), POL (9.1%), PPO (8.0%), LaOP (6.6%), POS (5.1%), and MOO (5.0%) are more abundant in HM; PUFA, highly unsaturated TG and linolenic acid are more abundant in DM.	[83]
Human milk, Bovine milk and Lacprodan	GC-MS/MSALL	152 lipids in 6 subclasses (16 FAs, TG, PC, PE, PS, PI, and SM). Lacprodan was similar to HM in FA, but was not detected in GP of short-chain FA.	[84]
Human milk, Bovine milk, n-3-enriched milk and Goat milk	HPLC-APCI-MS/MS	160 different TGs, MUFA (56.5%) and PUFA (30.8%) occurring in TG was a feature of HM, PUFA (C18:2, C18:2, DHA) appeared in n-3-enriched milk.	[16]
Human milk and Bovine milk	UPLC-Q-TOF-MS	215 potential biomarkers (72 PEs, 21 PGs, 4 PSs, 5 PIs, 18 PAs, 28 PCs, 6 CLs, 5 SMs, 32 HexCers, 12 DGs, and 13 TGs).	[79]
	UPLC-QTOF-MS	39 different lipids, 11 polar lipids (mainly SM), 3 HexCers, 12 DGs, and 13 TGs.	[74]
Human and Goat milk	UPLC-Q-TOF-MS	147 potential biomarkers (1 DG, 12 TGs, 16 PAs, 19 PCs, 54 PEs, 20 PGs, 7 PIs, 3 PSs, 4 CLs, 18 Hex2Cers, and 4 HexCers).	[79]
Human and Ewe milk	UPLC-QTOF-MS	14 different lipids, 1 PC (26:0/0:0), 1 DG, and 12 TGs.	[74]
Bovine and Ewe milk	UPLC-QTOF-MS	Three TG ((14:0/16:1/18:1), (16:0/16:1/18:3), (17:2/18:0/20:5)) with significant differences.	[74]
Bovine, Goat, and Sheep milk	HPLC-ELSD	BM was significantly higher in PE and lower in PC and SM, polar lipids (LacCer, PI) were higher in GM, and Chol+DG+FFA were lower in sheep milk.	[8]
Colostrum and milk (Bovine milk)	UHPLC-Q-TOF-MS	63 differential lipids, 4 potential biomarkers, PE (P-16:0/22:5), PE (P-16:0/20:4), Hex2Cer (d14:0/24:0), and Hex2Cer (d15:0/24:0).	[80]
Ultra-high temperature and Reconstituted milk (whole milk)	UPLC-Q-Exactive Orbitrap-MS	15 potential biomarkers, PE (16:0/18:1), PE (18:0/18:1), PE (18:1/18:1), PE (18:1/20:3), PE (18:1/14:0), TG (6:0/14:1/18:3), TG (15:0/14:0/10:0), PC (18:0/18:1), LPE (18:0), CL (18:1/18:0/18:0/18:1), 16-hydroxyhexadecanoic acid, 12-hydroxydecanoic acid, TG (16:0/8:0/18:1), TG (6:0/18:0/18:1), and PE (16:0/18:2).	[66]
Bovine milk, Goat milk, pasteurized bovine milk, pasteurized goat milk, bovine yogurt and goat yogurt	UHPLC-Q-Exactive-MS	1607 lipids species in 27 lipids subclasses, 27 potential biomarkers for pasteurized bovine and goat milk, and 23 potential biomarkers (7 PEs, 4 PLs, 4 PIs, 3 TGs, 2 SMs, 2 PSs and 1 PC) for bovine and goat’s yogurt.	[85]

Abbreviation: HM, human milk; BM, bovine milk; DM, donkey milk; GM, goat milk; IFM, infant formula milk; MUFA, monounsaturated FA; DGLA, dihomo-γ-linolenic acid; LA, linoleic acid; MCSAT, Medium-chain saturated fatty acids; LC-PUFAs, long-chain polyunsaturated fatty acids; PE-O, ether analog of glycerophoshoethanoloamine; POO, 1-palmitoylglycerol-2,3-dioleoyl; POL, 1-palmitoyl-2-oleoyl-3-linoleic; PPO, 1,2-dipalmitoyl-3-oleoylglycerol; LaOP, 1-lauric-2-palmitoylglycerol-3-oleoyl; POS 1-palmitic-2-oleoylglycerol-3-strearic; MOO, 1-myristic-2,3-dioleoyl. For the other abbreviations, see the footnote to Table 1.

## Data Availability

The data presented in this study are available on request from the corresponding author.
